# The safety attitudes questionnaire for out-of-hours service in primary healthcare—Psychometric properties of the Croatian version

**DOI:** 10.1371/journal.pone.0242065

**Published:** 2020-11-13

**Authors:** Jasna Mesarić, Diana Šimić, Milica Katić, Ellen Catharina Tveter Deilkås, Dag Hofoss, Gunnar Tschudi Bondevik

**Affiliations:** 1 Faculty of Health Sciences, Libertas International University, Zagreb, Croatia; 2 Croatian Society for Quality Improvement in Healthcare, Croatian Medical Association, Zagreb, Croatia; 3 Faculty of Organization and Informatics, University of Zagreb, Varaždin, Croatia; 4 School of Medicine, University of Zagreb, Zagreb, Croatia; 5 The Norwegian Directorate of Health, Oslo, Norway; 6 Health Services Research Unit, Akershus University Hospital, Lørenskog, Norway; 7 Lovisenberg Diaconal College, Oslo, Norway; 8 Department of Global Public Health and Primary Care, University of Bergen, Bergen, Norway; 9 National Centre for Emergency Primary Health Care, NORCE Norwegian Research Centre, Bergen, Norway; Universitat de Valencia, SPAIN

## Abstract

The aim of the study was to assess the reliability and construct validity of the Croatian translation of the Safety Attitudes Questionnaire—Ambulatory version (SAQ-AV) in the out-of-hours (OOH) primary care setting. A cross-sectional observational study using anonymous web-survey was carried out targeting a convenience sample of 358 health professionals working in the Croatian OOH primary care service. The final sample consisted of 185 questionnaires (response rate 51.7%). Psychometric properties were assessed using exploratory hierarchical factor analysis with Schmid-Leiman rotation to bifactor solution, McDonald’s ω, and Cronbach’s α. Five group factors were identified: Organization climate, Teamwork climate, Stress recognition, Ambulatory process of care, and Perceptions of workload. Items loading on the Stress recognition and Perceptions of workload factor had low loadings on the general factor. Cronbach’s α ranged between 0.79 and 0.93. All items had corrected item-total correlation above 0.5. McDonalds’ ω total for group factors ranged between 0.76 and 0.91. Values of ω general for factors Organization climate, Teamwork climate, and Ambulatory process of care ranged between 0.41 and 0.56. McDonalds’ ω general for Stress recognition and Perceptions of workload were 0.13 and 0.16, respectively. Even though SAQ-AV may not be a reliable tool for international comparisons, subsets of items may be reliable tools in several national settings, including Croatia. Results confirmed that Stress recognition is not a dimension of patient safety culture, while Ambulatory process of care might be. Future studies should investigate the relationship of patient safety culture to treatment outcome.

## Introduction

Patient safety is a major concern of health care services. The World Health Organisation defines patient safety as "the absence of preventable harm to a patient during the process of health care" [1]. Responsible health care organizations strive to avoid adverse events through improvement of the patient safety culture. The concept of “culture of safety” is multidimensional and comprises individual and group attitudes, beliefs, values, and competencies of health care professionals, staff interactions, clinical and administrative procedures, and practices aiming to protect patients from adverse events [2]. Lack of job satisfaction and work related stress might also increase the risk of human error in health care. Assessment of safety culture has been based on a variety of tools and instruments that have focused mainly on hospital care [3]. One of the most widely used instruments to measure safety culture of front line workers is the Safety Attitudes Questionnaire (SAQ) [2]. It has been validated in different languages and has shown good psychometric properties in different settings [4–13]. SAQ consists of 30 items arranged in six major patient safety subscales: Teamwork climate, Safety climate, Working condition, Job satisfaction, Perceptions of management, and Stress recognition [2].

The bulk of health care services is provided within the primary health care setting. However, concerns about patient safety in primary health care have been raised only recently. In 2007, Modak et al [14] extended SAQ with additional 32 items in order to adapt it to the primary care setting as Safety Attitude Questionnaire—Ambulatory Version (SAQ-A). In that study six SAQ subscales were confirmed for the original SAQ items. Additional subscale Ambulatory process of care was hypothesized, but not tested. In 2014, Bondevik et al [15] translated the SAQ-A into Norwegian and modified it by adding three other items from the SAQ-A to the Teamwork climate scale, and moving one item from the factor Perceptions of management to Working conditions. Bondevik et al [15] created two versions of the questionnaire—one for regular general practice, and one for out-of-hours (OOH) practice (used here as SAQ-AV) based on feedback from a group of primary health care providers. They used confirmatory factor analysis and confirmed five out of six SAQ subscales (all except Stress recognition) in the Norwegian setting. To distinguish it from Modak’s SAQ-A, we refer to it as SAQ-AV. SAQ-AV was subsequently translated into Dutch [16], and Slovenian [17], and applied to the out-of-hours primary health care services. In the Dutch and Slovenian settings these five subscales were not confirmed by confirmatory factor analysis. Authors of these studies performed exploratory factor analyses in order to explore dimensionality of SAQ-AV in their national settings [16, 17]

In Croatia, out-of-hours (OOH) primary health care services are provided by health care centres and emergency medical services. The 49 health care centres are primary health care organizations that provide general practice, dental, gynaecological, paediatric, and occupational healthcare, nursing and palliative care, laboratory and radiological diagnostics, and pharmacy services. Emergency medical services comprise first aid and emergency transport. OOH services in the health care centres are provided by general practitioners who usually rotate between their practice in normal office hours and OOH services. These services are available on weekends and holidays, but do not cover night shift. To our best knowledge patient safety culture in primary health care in Croatia has previously not been studied. A Croatian instrument for assessment of patient safety climate in primary care has not been developed until the Croatian translation of the SAQ-AV—the psychometric properties of which are reported here. The study was a part of the international study Patient Safety Culture in European Out-of-hours Services (SAFE-EUR-OOH) led by the Norwegian coordinating group of the European research network for out-of-hours primary health care (EurOOHnet) [18].

The aim of the study was to assess the reliability and construct validity of the Croatian version of the SAQ-AV.

## Materials and methods

### Ethics statement

The study was approved by the Ethical Committee of the School of Medicine, University of Zagreb (Case number 380-59-10106-15-168/120 of May 20, 2015). Data collection was compliant with Helsinki Declaration ethical guidelines. All participants received information about the purpose of the study, anonymity and confidentiality by e-mail. All participants gave an oral informed consent to participate in the study. Their participation was voluntary and data were analysed anonymously.

### SAQ translation

We have translated the English version of SAQ-AV [11] into Croatian. The translation procedure followed the principles adapted from Beaton et al. [19]. The English version of the SAQ-AV was translated into Croatian using a professional translation service. The translation was discussed in an expert committee of researchers and general practitioners and adapted to the Croatian OOH primary care setting. This adapted version was back-translated into English by an independent translation service blinded to the original version. The back-translation was compared with the original questionnaire in order to clarify possible misunderstandings. The final version was piloted on ten OOH general practitioners and was found to require no further modifications.

### Scoring

The questionnaire comprised 62 items [11] where study participants rated their agreement on a 5-point ordinal scale: 1 = disagree strongly, 2 = disagree slightly, 3 = neutral, 4 = agree slightly, 5 = agree strongly. All items also included the response option “Not applicable”–which was treated as missing data (non-response) in the data analyses. Scores of negatively worded items were reversed prior to analyses. Thus, higher scores indicate more positive evaluation of patient safety culture.

### Study design

A cross-sectional observational study using web-survey was carried out between June 29th and September 1st, 2015. We targeted the 29 largest (out of the total of 49) health care centres in Croatia providing general practice OOH services. According to publicly available statistics (http://www.hzzo.hr) these centres employ approximately 85% of all staff providing general practice OOH services. Recommendations for minimal sample size in factor analysis range from 3 to 10 participants per item [20]. Bujang et al [21] support the ratio 1:3 when other criteria of sampling adequacy are met, and the response scale has at least four levels. For our questionnaire with 62 five level items recommendations for a suitable sample size range between 186 and 620. We contacted all 358 health professionals with an e-mail address working in these health centres, and 185 returned the questionnaire (response rate 51.7%). Respondents who returned the questionnaire could have selected response NA to some of the items. Unanswered items and NAs were treated as missing data in the analysis.

### Statistical analysis

Statistical analysis included descriptive statistics (S1 Script), exploratory factor analysis (weighted least squares hierarchical factor analysis with oblimin rotation and Schmid-Leiman transformation), item-to-factor correlations, and intercorrelations of factors, measures of scale reliability (Cronbach’s α and McDonald’s ω hierarchical and total), and corrected item total correlation (CITC) (S3 Script) [22–26]. All analyses were done using the statistical software R version 3.2.2 (2015-08-14) [27], the RStudio (Version 0.99.486) [28] and the R package psych [29].

### Item selection

We used Kaiser-Meyer-Olkin (KMO) measure of sampling adequacy (MSA) to ensure that our correlation matrix was appropriate for factor analysis [30, 31]. In the first step we have excluded from the analysis items with proportion of response NA exceeding 9% (8 items). According to Kaiser [30] item level MSAi “measures to what extent a given variable belongs to the family, psychometrically.” Thus, item with MSAi below 0.7 were also excluded [20, 30–32]. Following factor analysis items with communality below 0.35 were excluded. Finally, CITC was used to check for homogeneity of items within the scale [33]. All retained items had CITC above 0.3. The number of items included in the final analysis was 37. (S2 Script).

### Exploratory factor analysis

If the Croatian version of SAQ-AV is a valid instrument for assessing overall patient safety attitudes in OOH primary care services, correlations between subscales could be attributed to a general factor representing this overall level of patient safety attitude. Therefore, we have used exploratory hierarchical factor analysis to identify the factor structure of the questionnaire and account for subscale intercorrelations. The goal of the factor analysis is to identify a smaller number of quantitative variables (factors) that explain a large proportion of the total variation of questionnaire items. In order to increase interpretability, obtained factors are rotated to achieve a simpler structure. Items loading on a single factor usually belong to the same subscale. When all items reflect a common underlying concept (like patient safety attitude) we may expect that a simple structure can only be achieved at the expense of factor independence (i.e. by non-orthogonal rotation). When rotated factors are correlated, a hierarchical (second order) factor analysis can be applied to these factors to obtain a common higher-order factor representing common variation in all items.

Exploratory hierarchical factor analysis (weighted least squares estimation) was used for exploring the dimensionality of the Croatian SAQ-AV. Factor analysis was done on the correlation matrix (Pearson r’s) with pairwise deletion of missing data. Correlation smoothing was used to achieve positive definiteness [34]. Horn’s [35] parallel analysis was used for selection of the number of factors. Oblimin rotation was used in order to obtain better separation of item loadings on factors and to estimate the inter-factor correlation matrix, which is the input for the second order factor analysis. We assumed that inter-factor correlations could be attributed to a general factor reflecting overall patient safety attitude. Therefore, the inter-factor correlation matrix was submitted to a second-order factor analysis extracting only one factor. Subsequently, Schmid-Leiman transformation of obtained factors was used to create an orthogonal solution with the general factor reflecting overall patient safety attitude, and group factors reflecting specific dimensions of patient safety culture and explaining the residual inter-item correlations after extraction of the general factor.

Reise et al. [26] compare three approaches to factor analysis of multidimensional constructs. The first one, most frequent in the literature, is correlated factors model, where factors are rotated using oblique rotation to achieve item separation and interpretability, allowing for correlation between subscales. However, as Reise et al. [26] highlight, this approach does not enable identification of a single common factor (the target construct) nor its direct association with items. The second one is hierarchical model, where correlations among factors are further submitted to factor analysis to identify a common second order factor. In this approach the single common factor (the target construct) is identified, however the relationship between an item and the target construct is “mediated through the primary factor” The approach we use is the third one, the bifactor model, where each item loads on the general factor and one or more group factors. According to Reise [36] in comparison to the first two approaches, the bifactor modelling enables “(a) partitioning item response variance into general versus group factor sources, (b) determining the degree to which item response data are unidimensional versus multidimensional, (c) estimating the degree to which raw scale scores reflect a single common source, and (d) evaluating the viability of subscale scores after variance due to the general factor has been controlled for.” We report results of Schmid-Leiman transformation without further selection of items to substantiate our conclusions regarding dimensionality of the concept of patient safety attitudes, and the viability of subscales.

In addition to factor loadings we report item communalities, (the proportion of item variance explained by the factor model), uniqueness (the proportion of item variance not accounted for), and the proportion of communality accounted for by the general factor [25, 26].

### Scale reliability

We use two conceptualizations of internal reliability. The first one is based on classical measurement theory where reliability is defined as a ratio of true score variance to total variance. The most popular representative of this conceptualization is Cronbach’s α that is based on average inter item covariance and represents “internal consistency” reliability [23]. The other conceptualization is based on the common factor model, where reliability is defined as a proportion of total score variance accounted for by common factors. We have used McDonald’s ω to estimate reliability of general factor, group factors and total reliability [23]. While Cronbach’s α assumes unidimensional concept, McDonald’s ω can be used as an estimate of general scale saturation for a multidimensional construct [22, 23, 37]. When an instrument is composed of two or more subscales, we would like to know whether it measures a unidimensional or a multidimensional construct. McDonald’s *ω total* is calculated from results of a hierarchical factor analysis as a ratio between variance accounted for by extracted general and group factors and the total variance. *ω general* is a ratio of the variance accounted for by the general factor and the total variance. *ω group* is a ratio of the variance accounted for by all group factors and the total variance. However, in calculating *ω group* variance accounted for by a group factor is taken only over items that load on that factor; thus, for the general factor, *ω general* and *ω group* do not have to add up to *ω total*. For each group factor *ω total*, *ω general* and *ω group* are calculated using only items that load on that factor. For a group factor *ω group* and *ω general* add up to *ω total*. The ratio of the *ω general* in relation to *ω total* provides a measure of general factor saturation. Higher values of *ω general* support unidimensionality, while lower values support multidimensionality of the measured construct. We also use CITC to identify any items that may lower reliability of a subscale.

## Results

The 185 respondents who completed the questionnaire came from 28 out of 49 health care centres in Croatia. Respondents included 150 (81.1%) medical doctors and 35 (18.9%) support medical staff (including registered nurses and administrative staff). The majority of respondents were female (84.3%). Thirty-seven (20%) of the respondents were under 30 years old, and six (3.2%) above 60 years. Respondents’ job experience in OOH service was below five years for 47 (25.4%) of the respondents. Job experience was uniformly distributed up to 40 years. Two respondents (1.1%) had more than 40 years of job experience. Only 86 (46.5%) respondents provided data on length of work in the OOH service where they were currently employed. Among those who responded, 32 (17.3%) had worked up to 10 years, 35 (18.9%) between 11 and 20 years, and 19 (10.3%) longer than 20 years in the current OOH service. The younger staff constituted 25% of our respondents (with less than 5 years of job experience). Most respondents (160, 86.5%) worked in shifts, while 18 (9.7%) of the respondents worked morning shift only (e.g. on Saturday and Sunday) and 7 (3.8%) worked afternoon shifts only.

Table 1 provides basic descriptive statistics for the scale items. For 15 out of the 62 items there were no missing data. The number of missing responses for other items ranged from 1 to 49. Items with the highest numbers of missing responses were SAQ42 (“Trainees in my discipline are adequately supervised”– 49 missing responses), SAQ50 (“Important issues are well communicated at shift changes”: 37 missing responses), and SAQ39 (“I am frequently unable to express disagreement with staff physicians/intensivists in this office”: 36 missing responses).

**Table 1:** Descriptive statistics for 62 items of the SAQ-AV questionnaire (185 respondents from 29 health care centres in Croatia providing out-of-hours services).

Item	NA^a^ n (%)	Mean score^b^ (SD^c^)
1. High levels of workload are common in this office.	0 (0.0%)	4.3 (1.0)
2. I like my job.	0 (0.0%)	4.8 (0.4)
3. Nurse input is well received in this office.	0 (0.0%)	4.6 (0.7)
4. I would feel safe being treated here as a patient.	0 (0.0%)	4.3 (0.9)
5. Medical errors are handled appropriately in this office.	5 (2.7%)	4.3 (0.8)
6. This office does a good job of training new personnel.	17 (9.2%)	3.7 (1.3)
7. All the necessary information for diagnostic and therapeutic decisions is routinely available to me.	4 (2.2%)	3.2 (1.4)
8. Working in this office is like being part of a large family.	1 (0.5%)	3.4 (1.4)
9. Senior management of this office is doing a good job.	9 (4.9%)	3.2 (1.2)
10. The management of this office supports my daily efforts.	11 (5.9%)	3.3 (1.2)
11. I receive appropriate feedback about my performance.	6 (3.2%)	2.8 (1.4)
12. In this office, it is difficult to discuss errors.	8 (4.3%)	2.9 (1.2)
13. Briefing other personnel before a procedure (e.g., biopsy) is important for patient safety.	23 (12.4%)	4.4 (0.9)
14. Briefings are common in this office.	20 (10.8%)	2.8 (1.4)
15. This office is a good place to work.	0 (0.0%)	3.8 (1.3)
16. Communication breakdowns which lead to delays in delivery of care are common.	3 (1.6%)	2.4 (1.2)
17. Office management does not knowingly compromise the safety of patients.	5 (2.7%)	3.0 (1.2)
18. The levels of staffing in this office are sufficient to handle the number of patients.	1 (0.5%)	4.2 (1.0)
19. Decision making in this office utilizes input from relevant personnel	0 (0.0%)	4.3 (0.9)
20. I am encouraged by my colleagues to report any patient safety concerns I may have.	6 (3.2%)	3.3 (1.2)
21. The culture in this office makes it easy to learn from the errors of others.	4 (2.2%)	3.4 (1.2)
22. This office deals constructively with problem personnel.	12 (6.5%)	3.0 (1.3)
23. The medical equipment in this office is adequate.	0 (0.0%)	3.2 (1.4)
24. In this office, it is difficult to speak up if I perceive a problem with patient care.	2 (1.1%)	2.6 (1.3)
25. When my workload becomes excessive, my performance is impaired.	1 (0.5%)	3.7 (1.3)
26. I am provided with adequate, timely information about events in the office that might affect my work.	6 (3.2%)	2.8 (1.3)
27. I have seen others make errors that had the potential to harm patients.	9 (4.9%)	3.1 (1.1)
28. I know the proper channels to direct questions regarding patient safety in this office.	6 (3.2%)	2.4 (1.3)
29. I am proud to work at this office.	1 (0.5%)	3.7 (1.2)
30. Disagreements in this office are resolved appropriately (i.e., not who is right but what is best for the patient).	5 (2.7%)	3.8 (1.1)
31. I am less effective at work when fatigued.	0 (0.0%)	3.8 (1.2)
32. I am more likely to make errors in tense or hostile situations.	0 (0.0%)	3.7 (1.1)
33. Stress from personal problems adversely affects my performance.	0 (0.0%)	2.7 (1.2)
34. I have the support I need from other personnel to care for patients.	0 (0.0%)	4.0 (1.0)
35. It is easy for personnel in this office to ask questions when there is something that they do not understand.	7 (3.8%)	4.2 (1.0)
36. Disruptions in the continuity of care can be detrimental to patient safety.	3 (1.6%)	4.4 (0.8)
37. During emergencies, I can predict what other personnel are going to do next.	1 (0.5%)	4.0 (0.9)
38. The physicians and nurses here work together as a well-coordinated team.	3 (1.6%)	4.2 (0.9)
39. I am frequently unable to express disagreement with staff physicians/intensivists in this office.	36 (19.5%)	2.6 (1.1)
40. Truly professional personnel can leave personal problems behind when working.	2 (1.1%)	4.2 (0.7)
41. Morale in this office is high.	3 (1.6%)	4.1 (0.9)
42. Trainees in my discipline are adequately supervised.	49 (26.5%)	3.9 (1.1)
43. I know the first and last names of all the personnel I worked with during my last shift.	8 (4.3%)	4.5 (1.0)
44. I have made errors that had the potential to harm patients.	1 (0.5%)	2.0 (1.2)
45. Attending physicians/primary care providers in this office are doing a good job.	8 (4.3%)	3.9 (0.9)
46. All the personnel in this office take responsibility for patient safety.	1 (0.5%)	4.3 (0.8)
47. I feel fatigued when I have to get up in the morning and face another day on the job.	1 (0.5%)	2.3 (1.3)
48. Patient safety is constantly reinforced as the priority in this office.	5 (2.7%)	4.0 (1.0)
49. I feel burned out from my work.	1 (0.5%)	3.0 (1.3)
50. Important issues are well communicated at shift changes.	37 (20.0%)	3.7 (1.2)
51. There is widespread adherence to clinical guidelines and evidence-based criteria in this office.	1 (0.5%)	3.9 (0.9)
52. I feel frustrated by my job.	0 (0.0%)	2.0 (1.2)
53. I feel I am working too hard on my job.	0 (0.0%)	3.4 (1.2)
54. Information obtained through incident reports is used to make patient care safer in this office.	29 (15.7%)	3.4 (1.3)
55. Personnel frequently disregard rules or guidelines (e.g., hand washing, treatment protocols/clinical pathways, sterile fields, etc.) that are established for this office.	2 (1.1%)	2.2 (1.1)
56. Fatigue impairs my performance during emergency situations (e.g. code or cardiac arrest).	10 (5.4%)	2.5 (1.4)
57. Fatigue impairs my performance during routine care.	0 (0.0%)	3.0 (1.3)
58. I am satisfied with the current referral process in this office.	0 (0.0%)	3.6 (1.2)
59. There is adequate and timely transfer of patient information between the primary care physician and the specialist.	2 (1.1%)	2.8 (1.3)
60. Medications are refilled in a timely manner.	4 (2.2%)	4.0 (1.1)
61. Medications are refilled correctly.	3 (1.6%)	4.1 (1.0)
62. Abnormal test results are frequently lost or overlooked.	18 (9.7%)	1.9 (1.0)

^a^ NA = not applicable

^b^ Scoring: 1 = Disagree strongly, 2 = Disagree slightly, 3 = Neutral, 4 = Agree slightly, 5 = Agree strongly

^c^ Standard deviation

Cronbach’s α for the entire set of 62 items was 0.95 (CI_95_ = 0.94–0.96). Corrected item-total correlations (CITC) ranged from 0.11 to 0.72 with the lowest three for items SAQ36 (“Disruptions in the continuity of care can be detrimental to patient safety” CITC = 0.11), SAQ27 (“I have seen others make errors that had the potential to harm patients” CITC = 0.16), and SAQ1 (“High levels of workload are common in this office” CITC = 0.17). Five items had CITCs between 0.2 and 0.3, all other items had CITCs above 0.3.

The overall Kaiser-Meier-Olkin measure of sampling adequacy (MSA) was 0.82, and MSA per item was above 0.5 for all items. Out of 62 items 6 had MSA values between 0.50 and 0.59, 8 had MSA between 0.60 and 0.69, 12 had MSA values between 0.70 and 0.79 and 36 had MSA of 0.8 and above.

The items with the lowest MSA values were SAQ1 (MSA = 0.53), SAQ36 (MSA = 0.55), and SAQ50 (MSA = 0.56).

**Fig 1:**
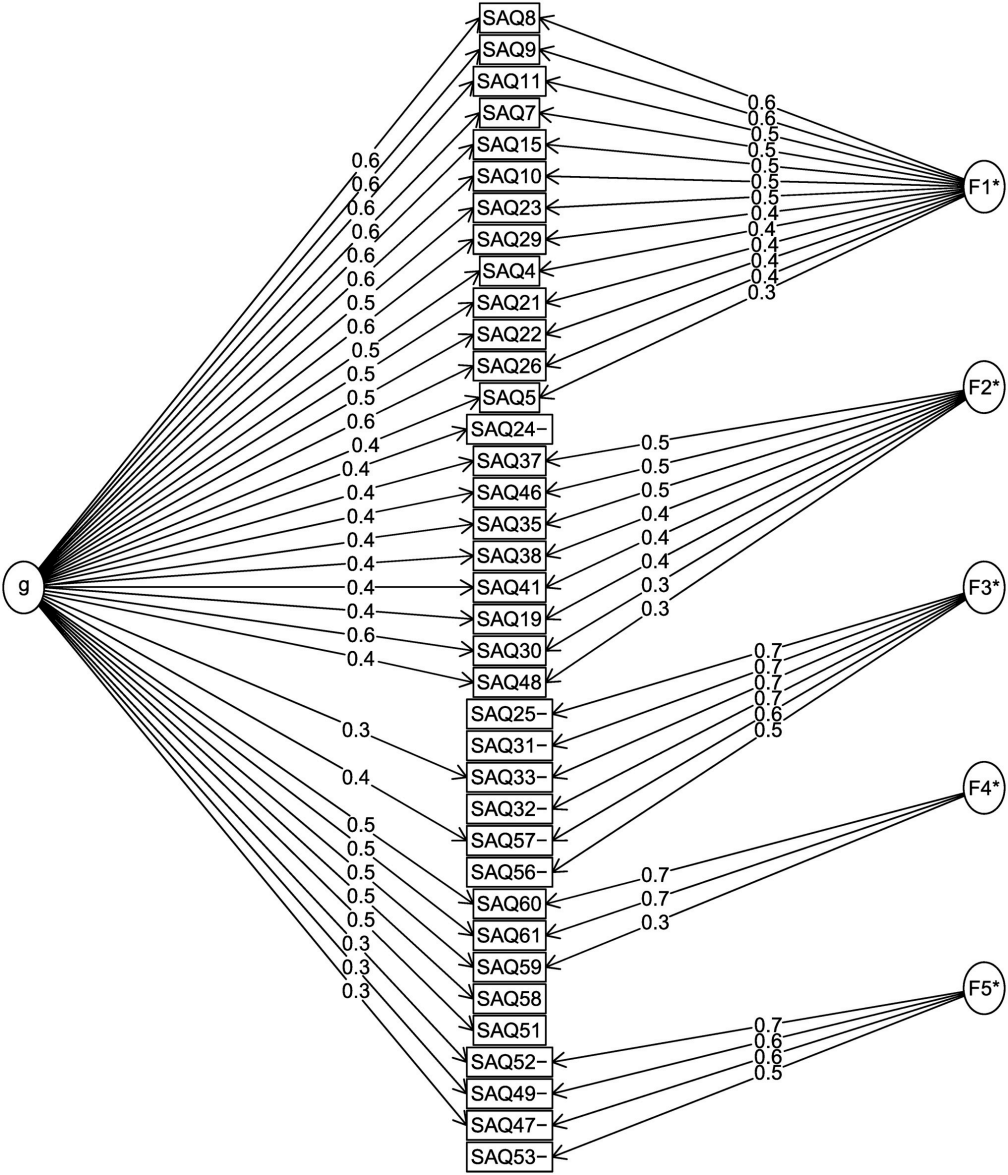
Factor structure model.

Exploratory hierarchical factor analysis was performed on the Pearson’s correlation matrix between 37 retained items, using pairwise deletion for items that included missing responses. Table 2 and Fig 1 present the structure of the final model. The five extracted factors accounted for 51.4% of the total variance. The proportion of explained common variance of the general factor was 42%, and the ratio between the eigenvalue corresponding to the general factor and the largest eigenvalue of group factors was 2.67. Items loading on the third and the fifth factor had low loadings on the general factor indicating that these factors were possibly not related to the patient safety culture attitude. The proportion of communality accounted for by the general factor for items loading on the third and the fifth factor was below 23%, while for items loading on other factors it ranged between 35% and 66%, with a median proportion of 53%. Three items had loading above 0.3 on the general factor, and the highest group factor loading below 0.3. Since their communalities were above 0.3, they were retained in the analysis.

**Table 2:** Schmid-Leiman factor loadings (g—general factor, F1* to F5* group factors), communality (h2), uniqueness (u2) and proportion of communality accounted for by the general factor (p2).

Item	g	F1*	F2*	F3*	F4*	F5*	h2	u2	p2
**SAQ4**	0.52	0.39					0.45	0.55	0.59
**SAQ5**	0.44	0.31					0.38	0.62	0.52
**SAQ7**	0.56	0.53					0.64	0.36	0.49
**SAQ8**	0.56	0.57					0.67	0.33	0.47
**SAQ9**	0.63	0.56					0.75	0.25	0.53
**SAQ10**	0.63	0.47					0.67	0.33	0.59
**SAQ11**	0.58	0.54					0.61	0.39	0.54
**SAQ15**	0.63	0.51					0.68	0.32	0.58
**SAQ19**	0.39		0.36				0.32	0.68	0.48
**SAQ21**	0.48	0.37					0.48	0.52	0.48
**SAQ22**	0.54	0.37					0.56	0.44	0.52
**SAQ23**	0.52	0.46					0.50	0.50	0.55
**SAQ24-**	0.43	0.25					0.32	0.68	0.58
**SAQ25-**				0.71			0.59	0.41	0.10
**SAQ26**	0.57	0.37					0.49	0.51	0.66
**SAQ29**	0.63	0.42					0.65	0.35	0.62
**SAQ30**	0.60		0.33				0.57	0.43	0.63
**SAQ31-**				0.69			0.56	0.44	0.13
**SAQ32-**				0.66			0.51	0.49	0.11
**SAQ33-**	0.32			0.66			0.56	0.44	0.18
**SAQ35**	0.42		0.46				0.45	0.55	0.39
**SAQ37**	0.41		0.52				0.45	0.55	0.38
**SAQ38**	0.44		0.43				0.40	0.60	0.49
**SAQ41**	0.44		0.41				0.39	0.61	0.50
**SAQ46**	0.40		0.48				0.43	0.57	0.38
**SAQ47-**	0.32					0.62	0.55	0.45	0.18
**SAQ48**	0.43		0.32				0.34	0.66	0.54
**SAQ49-**	0.33					0.63	0.54	0.46	0.20
**SAQ51**	0.47				0.28		0.40	0.60	0.56
**SAQ52-**	0.30					0.71	0.62	0.38	0.15
**SAQ53-**						0.46	0.38	0.62	0.23
**SAQ56-**				0.54			0.37	0.63	0.19
**SAQ57-**	0.35			0.61			0.58	0.42	0.21
**SAQ58**	0.47				0.29		0.37	0.63	0.58
**SAQ59**	0.45				0.35		0.37	0.63	0.55
**SAQ60**	0.53				0.72		0.80	0.20	0.35
**SAQ61**	0.52				0.67		0.73	0.27	0.37

General factor loadings less than 0.3 in absolute value and group factor loadings less than 0.28 in absolute value are not shown. Reversed items are marked with a “-” following their label.

Value of *ω total* for the general factor was 0.96, *ω general* was 0.66, and *ω group* was only 0.18. Values of *ω total* for group factors ranged between 0.76 and 0.91. Values of *ω general* for group factors Organization climate, Teamwork climate, and Ambulatory process of care ranged between 0.41 and 0.56, and for factors Stress recognition and Perceptions of workload they were 0.13 and 0.16, respectively. Values of *ω group* for factors Organization climate, Teamwork climate, and Ambulatory process ranged between 0.35 and 0.36, for factors Stress recognition and Perceptions of workload they were 0.70 and 0.61, respectively.

Table 3 shows the distribution of items in respect to the original SAQ-A subscales [14] and our group factors. Factors 2 to 4 can be easily identified with subscales Teamwork climate (F2*), Stress recognition (F3*), and Ambulatory process of care (F4*). The first factor (F1*) combines items from Safety climate, Job satisfaction, Perceptions of management, and Working condition subscales. We have named this factor Organization climate [38]. Factor 5 comprises items 47, 49, 52, and 53, added to the scale by Bondevik et al [15] (see Table 1 for item content). We have named this factor Perceptions of workload, as these items relate to “fatigue”, being “burned out”, and “work(ing) too hard”. All items correlated more strongly with their own factor than with other factors.

**Table 3:** Distribution of items on the five group factors and the original subscales of SAQ-A [14].

Original SAQ-A subscale	Factor					Total items
	F1*	F2*	F3*	F4*	F5*	
**Safety climate**	4					4
**Job satisfaction**	3	1				4
**Perceptions of management**	3					3
**Working condition**	2					2
**Teamwork climate**	1	3				4
**Stress recognition**			4			4
**Ambulatory process of care**				4		4
**Others**	1	3	1	1		6
**Total items**	14	7	5	5	0	31

Blank cells represent 0 items.

Table 4 presents values of Cronbach’s α and corrected item total correlation for group factors. All five subscales had Cronbach’s α’s greater than 0.79. Corrected item total correlations ranged between 0.53 and 0.81 for the first factor, between 0.56 and 0.69 for the second factor, between 0.62 and 0.77 for the third factor, between 0.56 and 0.88 for the fourth factor, and between 0.52 and 0.74 for the fifth factor. Croatian translation of items is provided in (S1 Text).

**Table 4:** Internal consistency of five group factors—Cronbach’s α and corrected item total correlations (CITC).

Item		CITC^a^
**Group factor 1 –Organizational climate** Cronbach’s α = 0.93		
**SAQ4**	I would feel safe being treated here as a patient.	0,67
**SAQ5**	Medical errors are handled appropriately in this office.	0,58
**SAQ7**	All the necessary information for diagnostic and therapeutic decisions is routinely available to me.	0,77
**SAQ8**	Working in this office is like being part of a large family.	0,79
**SAQ9**	Senior management of this office is doing a good job.	0,79
**SAQ10**	The management of this office supports my daily efforts.	0,75
**SAQ11**	I receive appropriate feedback about my performance.	0,78
**SAQ15**	This office is a good place to work.	0,81
**SAQ21**	The culture in this office makes it easy to learn from the errors of others.	0,66
**SAQ22**	This office deals constructively with problem personnel.	0,73
**SAQ23**	The medical equipment in this office is adequate.	0,70
**SAQ24-**	In this office, it is difficult to speak up if I perceive a problem with patient care.	0,53
**SAQ26**	I am provided with adequate, timely information about events in the office that might affect my work.	0,68
**SAQ29**	I am proud to work at this office.	0,78
**Group factor 2 –Teamwork climate** Cronbach’s α = 0.84		
**SAQ19**	Decision making in this office utilizes input from relevant personnel	0.57
**SAQ30**	Disagreements in this office are resolved appropriately (i.e., not who is right but what is best for the patient).	0.68
**SAQ35**	It is easy for personnel in this office to ask questions when there is something that they do not understand.	0.59
**SAQ37**	During emergencies, I can predict what other personnel are going to do next.	0.69
**SAQ38**	The physicians and nurses here work together as a well-coordinated team.	0.69
**SAQ41**	Morale in this office is high.	0.56
**SAQ46**	All the personnel in this office take responsibility for patient safety.	0.65
**SAQ48**	Patient safety is constantly reinforced as the priority in this office.	0.58
**Group factor 3 –Stress recognition** Cronbach’s α = 0.86		
**SAQ25-**	When my workload becomes excessive, my performance is impaired.	0.74
**SAQ31-**	I am less effective at work when fatigued.	0.73
**SAQ32-**	I am more likely to make errors in tense or hostile situations.	0.67
**SAQ33-**	Stress from personal problems adversely affects my performance.	0.74
**SAQ56-**	Fatigue impairs my performance during emergency situations (e.g. code or cardiac arrest).	0.62
**SAQ57-**	Fatigue impairs my performance during routine care.	0.77
**Group factor 4 –Ambulatory process of care** Cronbach’s α = 0.82		
**SAQ51**	There is widespread adherence to clinical guidelines and evidence-based criteria in this office.	0.56
**SAQ58**	I am satisfied with the current referral process in this office.	0.60
**SAQ59**	There is adequate and timely transfer of patient information between the primary care physician and the specialist.	0.64
**SAQ60**	Medications are refilled in a timely manner.	0.88
**SAQ61**	Medications are refilled correctly.	0.83
**Group factor 5 –Perceptions of workload** Cronbach’s α = 0.79		
**SAQ47**	I feel fatigued when I have to get up in the morning and face another day on the job.	0.72
**SAQ49**	I feel burned out from my work.	0.74
**SAQ52**	I feel frustrated by my job.	0.72
**SAQ53**	I feel I am working too hard on my job.	0.52

^a^ corrected item total correlation

## Discussion

Exploratory bifactor analysis of the Croatian version of the SAQ-AV revealed five factors. These factors partially corresponded to previously identified SAQ factors in other countries [2, 14–17]. Separation of items is very good, i.e. items do not have high loadings on more than one factor, confirming discriminant validity of identified subscales. Based on values of *ω* general, items loading on factors Organization climate, Teamwork climate, and Ambulatory process reflect a common construct, while items loading on factors Stress recognition and Perceptions of workload do not. Relatively lower values of *ω* group for the first three factors do not support conclusion that these factors reflect viable subscales after accounting for item variability due to the general factor.

The first extracted factor cannot be clearly mapped to any single SAQ-A subscale [11]. It comprises items belonging to the subscales Safety climate (4 items), Job satisfaction (3 items), Perceptions of management (3 items), Working condition (2 items), and Teamwork climate (1 item). We believe that this can be attributed to characteristics of the OOH services in Croatia, where these items reflect aspects of organization climate, which combines these four original dimensions [38]. This factor is highly reliable with Cronbach α of 0.93 and ω total of 0.91. It’s ω general is 0.56, reflecting high common variation with the general factor.

The second factor corresponds to the SAQ-A Teamwork climate subscale. It has reasonably high values of Cronbach’s α (0.84) ω total (0.77), and ω general (0.41), demonstrating its reliability.

The third factor is clearly defined by Stress recognition items. In comparison to SAQ-A subscales, this factor has the most consistent structure. It has relatively high values of Cronbach’s α (0.86) and ω total (0.84). However, it only marginally contributes to the general factor, with ω general equal only 0.13. According to Saxton et al [2] development of the SAQ questionnaire relied on “Vincent’s framework for analysing risk and safety, and Donabedian’s conceptual model for assessing quality”. Pool of items was generated through brainstorming with healthcare providers, and resulted in a six factor model including stress recognition as one of dimensions. However, subsequent studies showed low test-retest reliability, low correlation between stress recognition and other dimensions, and lack of variability in stress recognition where other dimensions showed variation among sites [4, 6, 9, 15, 17, 39, 40] Thus, our results confirm that Stress recognition is not a sub-dimension of patient safety culture attitude. We agree with Taylor and Pandian [41] that Stress recognition is an important standalone construct, however it does not reflect the safety climate.

Modak et al. [14] added “5 items pertaining to the process of ambulatory care based on literature review of outpatient medical errors, quality improvement, and patient safety” to the original SAQ. Their confirmatory factor analysis aimed only to confirm the six factor structure of the original SAQ; thus, “Ambulatory Process of Care Items” were not included in the factor analysis. Our factor four undoubtedly represents SAQ-A Ambulatory Process of Care [14], with four out of its five items belonging to this group of items. This factor exhibits reasonable reliability with Cronbach’s α of 0.82, and ω total of 0.77. Its ω general is 0.41, confirming further that this factor contributes to the general safety attitude scale.

Factor five comprises four items that relate to fatigue, frustration, burn-out, and perception of working too hard. These items differ from items on the Stress recognition subscale in that they relate to perception of personal workload, and not recognition of relationship between stress and performance. We named this factor Perceptions of workload. Even though this factor shows reasonable reliability (Cronbach’s α 0.79, ω total 0.76), it only marginally contributes to the general factor (ω general 0.16), which suggests that it may not be a reliable indicator of safety culture in Croatia. This may be due to a prevalent lack of staff in Croatian health care, reflected also in the fact that 55.7% of respondents agreed slightly or strongly with the item SAQ53 (“working too hard”).

Survey and item level non-response could have an impact on the results of an exploratory factor analysis. We had relatively low response rate of 51.7%. This could influence results if non-response was associated with patient safety climate. In such a case, we would expect to see skewed item distributions, due to lack of extreme responses in either positive or negative direction. However, none of the items in our analysis had skewness larger than 2 in absolute value, thus we believe that self-selection did not have undue influence on our results [42].

Regarding the item-level non-response, we decided to exclude from the analysis eight items with item non-response above 9%. These items can be classified in four groups. The first group (items SAQ6 and SAQ42) relates to training of new personnel and trainees. The second group were items regarding briefings and shift hand-overs (SAQ13, SAQ14, and SAQ50). The third group was related to incidents and incident reporting (SAQ54, SAQ62), and the last item was SAQ39 (inability to express disagreement). Non-response for items in groups one to three can be attributed to the organization of Croatian GP OOH (small number of trainees / new staff—thus rare organization of trainings, one-shift daily—thus no briefings and hand-overs, and underdeveloped incident reporting system). Regarding non-response for item SAQ39, while we have targeted the largest GP OOH, in some of them OOH team may only comprise one physician / intensivist, and for them there would be nobody to express disagreement with. We believe that these items do not reflect well safety climate attitudes in the Croatian setting, and that is why we decided to exclude the items, instead of imputing the missing data. Still, it would be interesting to analyse association between the non-response to these items and other responses, however that analysis is out of the scope of this paper.

It is interesting to compare our results with similar studies in other countries. In comparison to SAQ-A [14], there is a good correspondence between two of our factors with respective SAQ-A subscales (Stress recognition, and Teamwork climate). The original SAQ-A subdimensions Safety climate, Perceptions of management, Working condition, and Job satisfaction combined in a single factor we named Organization Climate.

Organization of the Croatian health care system is more similar to the Slovenian health care system than to other countries in which research on psychometric qualities of SAQ in primary care has been published so far. Due to similarities in organization of OOH services we would expect to find similarities with factor structure in Slovenia [17], however we did not.

Overall, only the Teamwork climate factor showed consistent presence in this and the three studies of SAQ in OOH primary care services in other European countries (Norway, Netherlands, and Slovenia) [15–17]. We have identified the Ambulatory process of Care factor as proposed in the original SAQ-A study [14] (USA). Stress recognition and Perceptions of workload were identified as reliable scales. They had Cronbach’s α equal 0.86 and 0.79, respectively, and their items did not cross-load on other (sub)scales. However, these scales are not subscales of the perception of safety culture, because their respective ω general were only 0.13 and 0.16. Perceptions of workload is a factor recognized in this study, that was not previously identified.

## Conclusions

Based on relatively high reliability of our factors, we believe that it would be possible to use the items loading on our factors Organization climate, Teamwork climate, and Ambulatory process as a reliable tool for measuring patient safety culture in Croatian OOH primary healthcare. Our results do not support use of raw subscale scores. A larger study is needed to confirm this. However, this tool may not be appropriate for international comparison. We believe that due to national peculiarities of the organization of health care the factor structure of patient safety culture measurements also varies across countries. For international comparisons item level comparison may be the best option. Our analysis did not find that Stress Recognition and Perception of workload were a sub-dimension of safety attitude in the Croatian primary care OOH service.

Future research is needed to understand better how differences in national health systems affect the structure of patient safety culture and elucidate the relationship between patient safety culture and treatment outcomes.

## Supporting information

S1 Script Descriptive statistics. (R)

S2 Script Item selection procedure. (R)

S3 Script Exploratory factor analysis. (R)

S1 Text Questionnaire in Croatian. (TXT)

## Abbreviations

EurOOHnet: European research network for out-of-hours primary health care
MSA: Measure of sampling adequacy
OOH: Out-of-hours (service)
SAFE-EUR-OOH: Patient Safety Culture in European Out-of-hours services
SAQ: Safety attitudes questionnaire
SAQ-A: Safety attitudes questionnaire—ambulatory version (Modak [14])
SAQ-AV: Safety attitudes questionnaire—ambulatory version (Bondevik et al [11, 15])
